# Neonatal macaque model to study *Mycobacterium tuberculosis* infection in pediatric AIDS

**DOI:** 10.1186/1471-2334-14-S2-O11

**Published:** 2014-05-23

**Authors:** Marie-Claire Gauduin, Magdalena Cepeda, Mary Salas, Robert White, Melissa de la Garza, Edward J Dick, Michael Owston, Lisa Y Armitige

**Affiliations:** 1 Texas Biomedical Research Institute, San Antonio, Texas, USA

## Introduction

Tuberculosis (TB) is the leading cause of death in AIDS patients worldwide; the earliest opportunistic infection occurring in conjunction with HIV; an accelerant of HIV replication and immune system deterioration. Co-infection with *Mycobacterium tuberculosis* (Mtb) and HIV is an increasing global emergency. Little is known regarding the early events of Mtb infection in humans especially infants. There is an urgent need for a clinically relevant animal model that mimics TB disease in human children to better understand early immune responses, pathogenicity and disease progression in newborn/infants.

## Methods

Aerosolized Mtb transmission was performed in neonatal macaques to mimic mother-to-child transmission and to: i. Characterize early TB-specific immune responses during acute Mtb-infection, ii. Investigate the dynamics of Mtb-specific T cell responses following early infection in infants; and, iii. Characterize the distribution and frequency of Mtb-specific responses in various host tissues. Six newborn macaques (6 weeks old) were infected via broncho or aerosol routes of infection with various Mtb doses (Erdman or Rh37rv strains). All clinical, immunologic, microbiologic, and pathologic events were assessed for 84 days post-infection.

## Results

Gross pathological abnormalities were observed as early as 35 et 42 days post infection including Ghon complex formation and granulomas in the lung. Caseous granulomas were also observed in the lungs at these early time-points, reflecting strong initial responses. Using ELISPOT assays, we found that IL-12 production correlated with early Mtb infection lesions seen by routine thoracic radiographs. Flow cytometry revealed robust granzyme B productions by NK8/NK cells that increased over time and transient Perforin productions that peak at day 56. Mtb-specific T cells responses were also detected and increased IL-12 and MIP-1β production in PBMC, Spleen and LN collected day 84.

## Conclusions

Overall, we successfully developed/optimized an aerosol neonatal macaque model that mimics clinical and bacteriological characteristics of early Mtb-infection as seen in human newborns/infants. This model represents a unique opportunity to characterize neonatal Mtb infection and, further understand all interactions between TB and HIV co-infection in pediatric AIDS and develop appropriate therapeutic interventions.

